# Periodontitis in obese adults with and without metabolic syndrome: a cross-sectional study

**DOI:** 10.1186/s12903-023-03133-5

**Published:** 2023-07-01

**Authors:** Astrid Nilsen, Anette Thorsnes, Stein Atle Lie, Paal Methlie, Dagmar F. Bunaes, Karen K. Reinholtsen, Knut N. Leknes

**Affiliations:** 1 Department of Clinical Dentistry – Periodontics, Faculty of Medicine, University of Bergen, Aarstadveien 19, Bergen, N-5009 Norway; 2 Department of Medicine, Haukeland University Hospital, Bergen, Norway; 3 Department of Clinical Science, University of Bergen, Bergen, Norway

**Keywords:** Cross-sectional study, Periodontitis, Obesity, Systemic mediators

## Abstract

**Background:**

Epidemiological studies support an association between obesity, metabolic syndrome (MetS), and periodontitis. Still, understanding of the effects of low-grade inflammation in obese subjects on periodontitis and influence of MetS remains incomplete. The aims of this cross-sectional study were to explore the association between obesity related variables and periodontitis, and assess if MetS is a risk indicator for periodontitis in a sample of obese adults.

**Methods:**

The study sample comprised 52 adults with a body mass index (BMI) of ≥ 30 kg/m^2^ referred for obesity therapy at the Obesity Centre at Haukeland University Hospital (HUH), Bergen, Norway. The subjects had prior to enrolment completed a 5-month lifestyle intervention course as part of a 2-year managing program. According to the revised National Cholesterol Education Program Adult Treatment Panel III (NCEP ATP III) classification of MetS, 38 subjects were recruited to the MetS group and 14 subjects to the non-MetS group. Medical data including peripheral blood samples were obtained from records at HUH at the time of enrolment. Probing depth, clinical attachment level, tooth mobility, furcation involvement, bleeding on probing (BoP) were recorded and intraoral bitewings evaluated at a full-mouth periodontal examination. Associations between risk variables for obesity/MetS and periodontitis were explored using linear and logistic regression models.

**Results:**

In the present sample 79% of the subjects were diagnosed with periodontitis. The prevalence of stage III/IV periodontitis was 42.9% in the non-MetS group vs. 36.8% in the MetS group (p = 0.200). In the non-MetS group 29.8% of the sites displayed BoP vs. 23.5% in the MetS group (p = 0.048). For stage III/IV periodontitis, the effect of age appeared to be significant for obesity related variables and MetS (p = 0.006, p = 0.002, respectively). None of the other analyses showed significant association with the outcome variables.

**Conclusion:**

In the present sample of obese subjects, periodontitis occurred independently of MetS. Reaching a certain BMI level, suggested association between MetS and periodontitis might be non-significant due to the dominating impact of obesity related variables undermining the effect of other systemic factors.

**Trial registration:**

The principal clinical trial, entitled “Obesity and Oral Diseases”, was prospectively registered in ClinicalTrials.gov with registration NCT04602572 (20.10.2020).

## Background

Overweight and obesity increasing globally are considered major challenges to human health services [1]. In 2016, 39% of adults were overweight and 13% were obese [2]. Present understanding recognizes adipose tissue as an endocrine organ, adipocytes acting as modulators affecting local and systemic inflammation. This low-grade inflammation may contribute to the occurrence of diabetes mellitus, cardiovascular disease, hypertension, stroke, and cancers [3, 4]. Several oral health concerns including periodontitis, tooth loss, xerostomia, dental erosion, and dental caries have also been linked with obesity [5].

Metabolic syndrome (MetS) represents an assembly of functional disturbances including obesity, increased waist circumference, increased insulin levels/blood glucose, hypertension and/or low high-density lipoprotein (HDL)/high triglycerides (TG) count. Each is considered a risk factor for the development of other diseases [6]. Co-occurring as a syndrome, these factors significantly increase risk of developing potentially life-threatening diseases [7]. The prevalence of MetS is estimated to be about 25% of the adult world population [8]. Age, familial history of type 2 diabetes, previous gestational diabetes, gout, and polycystic ovary syndrome are risk factors associated with increased risk of developing MetS [7]. There are several definitions of MetS, the revised version of the National Cholesterol Education Program Adult Treatment Panel III (NCEP ATP III) definition from 2005 was used in the present study [9].

Periodontitis is a chronic non-communicable, inflammatory disease encompassing tooth supporting gingival tissues and alveolar bone triggered by dysbiosis of oral microbiota and host immune response [10]. Periodontitis affects oral function and aesthetics impacting individual quality of life and well-being. Severe periodontitis is the sixth most prevalent non-communicable condition affecting approximately 10% of the world population [11].

Elevated levels of inflammatory biomarkers including high-sensitivity C-reactive protein (CRP) [12] and proinflammatory cytokines [13] have been observed in obese subjects. The association between obesity and periodontitis has been shown in several studies [14, 15]. However, the understanding of effects of low-grade inflammation in overweight/obese subjects on periodontitis remains incomplete [16]. A recent population-based cohort study reported that obese subjects presented a significantly higher risk of tooth loss than normal-weight subjects and that preventive strategies directed to reduce tooth loss should target obese subjects [17]. An obesity modulated imbalance may result in an increased expression of pro-inflammatory and reduction of anti-inflammatory adipokines, leading to a persistent chronic inflammatory status. The presence of insulin resistance and obesity might have a close relation to the plasma levels of TNF-α, CRP, and IL-6 [18].

Periodontitis and MetS share inflammation as a common feature and subjects affected by either disease may display elevated levels of circulatory cytokines. Both TNF-α and IL-6 have been considered important mediators for periodontitis [19]. Increased levels of these mediators in MetS subjects, and their association with development of MetS associated morbidities further support theories of a bidirectional relationship between MetS and periodontitis [15, 20]. Oxidative stress as a potential link between MetS and periodontitis has also received attention [21].

The aims of the present cross-sectional study were to explore the association between obesity related variables and periodontitis, and assess if MetS is a risk indicator for periodontitis in a sample of obese adults. We hypothesize that MetS deteriorates the periodontal inflammatory status in these subjects.

## Methods

The study protocol and informed consent following the Helsinki Declaration of 1975 (version 2008) was approved by the Norwegian Regional Ethics Committee (Reference 152,810–22/02/2021/REK Vest). The principal clinical trial, entitled “Obesity and Oral Diseases”, was prospectively registered in ClinicalTrials.gov with registration NCT04602572 (20.10.2020). Study subjects were informed of the nature, scope, and consequences of participating in the study before signing the informed consent. The present article is written according to Strengthening the Reporting of Observational Studies in Epidemiology (STROBE) guidelines. All methods were carried out in accordance with relevant guidelines and regulations.

### Study design and setting

The present cross-sectional study enrolled subjects from the Obesity Centre, Section of Endocrinology, Haukeland University Hospital (HUH), Bergen, Norway, January 2021 through September 2021. Medical data and fasting blood samples were collected at enrolment. A clinical periodontal examination was performed at the Department of Clinical Dentistry, University of Bergen, January 2021 through December 2021. Enrolled subjects were informed of any periodontal pathology and recommended to seek dental care from a local dentist or periodontal specialist of choice.

### Pre-study calibration and sample size calculation

One examiner (AT) unaware of group assignment performed all clinical examinations. Prior to initiation of the study, two separate calibration exercises were performed: 1: Examiner AT was calibrated with an experienced periodontist (KNL) and 2: Examiner AT performed an exercise to assess intra-examiner reproducibility for the primary outcome variables probing depth (PD) and clinical attachment level (CAL). In a study sample of five subjects (100 teeth/600 sites), PD and CAL were examined twice, 10 min apart, at six sites per tooth. Intraclass correlation coefficients (ICCs) were calculated separately for each site. ICC for repeated measures ranged between 0.84 and 0.93 for PD and between 0.82 and 0.95 for CAL.

The pre-study sample size calculation was based on change in PD. A difference of 0.5 mm was considered clinically relevant [22]. Standard deviation of the difference between repeated PD measurements from the intra-calibration exercise was 0.5 mm. A power analysis based on 50 subjects (25/group) with the level of significance (α) set to 0.05, resulted in 90% power to detect a true difference of 0.5 mm. Drop-outs were not anticipated.

### Study subjects

**Fig. 1:**
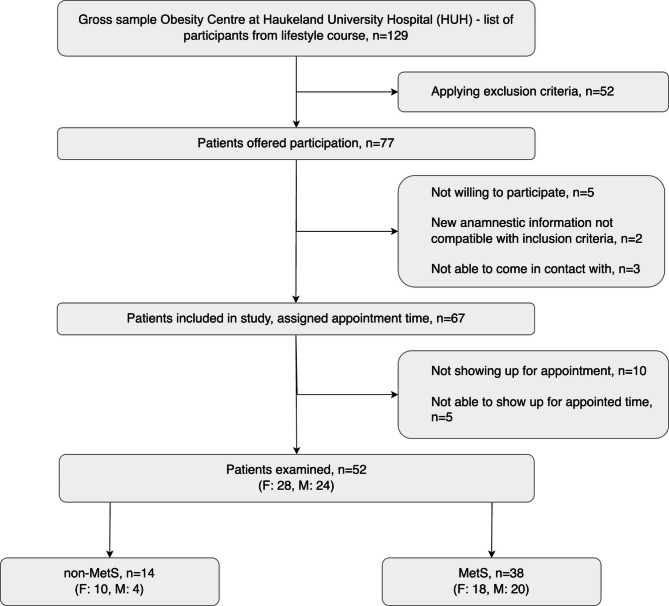
Flowchart of study participants

The study included subjects with an initial body mass index (BMI) ≥ 30 kg/m^2^ having completed a 5-month lifestyle intervention course as part of a 2-year managing program. Consecutive subjects were screened for eligibility according to inclusion/exclusion criteria by a senior consultant. Fifty-two out of 129 screened subjects were enrolled (Fig. 1).

Study exclusion criteria included leukaemia/neutropenia, HIV/AIDS, pregnancy, use of immunosuppressive drugs, mental illness (active psychosis, severe depression), Sjögren’s syndrome, obesity caused by endocrine disease (e.g., hypercortisolism, hypothyroidism), bulimia nervosa, and present/past history of head and neck cancer. In addition, subjects treated for periodontitis or used systemic antibiotic/anti-inflammatory medication within the last 6 months were also excluded.

Enrolled subjects were assigned into a non-MetS or a MetS group (Fig. 1) according to the revised NCEP ATP III classification [9]. MetS was determined when participants met at least three out of five criteria: waist circumference ≥ 102/88 cm, men/women; fasting triglycerides ≥ 1.7 mmol/L or drug treatment for elevated levels; HDL-cholesterol < 1.03/1.30 mmol/L, men/women; systolic blood pressure > 130 mmHg an/or diastolic blood pressure > 85 mmHg or antihypertensive medication; elevated fasting s-glucose ≥ 5.7 or anti-diabetic medication. Data on waist circumstance (WC) were missing, but WC was safely assumed to exceed the revised NCEP ATP III criteria in patients with BMI > 30 kg/m^2^. The blood sample for testing MetS diagnosis parameters (triglycerides, HDL-cholesterol, and fasting glucose) and CRP were all collected at HUH in the morning after 12 h fasting. Subjects were excluded if group allocation was not possible based on predefined MetS criteria.

### Questionnaire

Upon arrival for the clinical examination, study subjects completed a multiple-choice questionnaire including demographic items, general health, medication, smoking, oral health/hygiene, and dietary habits. Questions were repeated orally if there were missing answers resulting in a 100% response rate. The questionnaire, tested internally to ascertain that all items were easily understood, was constructed by the research group experienced in the items selected. Participants were classified as either current smokers (had smoked ≥ 100 cigarettes in their lifetime and currently smoked), former smokers (had smoked ≥ 100 cigarettes in their lifetime, not currently smoking), or never smokers (had not smoked ≥ 100 cigarettes in their lifetime) [23].

### Periodontal examination

The following clinical parameters were recorded: PD (distance in mm from the gingival margin to the base of the periodontal pocket); and CAL (distance in mm from the cemento-enamel junction or the margin of a dental restoration to the base of the pocket) using a periodontal probe (PCPUNC 15, Hu-Friedy, Chicago, IL, USA) at six sites per tooth rounded up to the nearest mm (mesio-buccal, buccal, disto-buccal, disto-palatal/lingual, lingual/palatal, mesio-lingual/palatal). Bleeding on probing (BoP), sites showing bleeding on gentle probing was estimated at four sites per tooth (mesial, buccal, distal, lingual) [24]. Furcations were examined using a curved Nabers furcation probe (PQ2N; HU-Friedy, Chicago, IL, USA) and recorded according to horizontal classification criteria [25]: Furcation defects, featuring loss of periodontal support > 3 mm into the furcation but not encompassing the total width of the furcation area were classified as degree II. Physiological tooth mobility was classified as degree 0, horizontally mobility < 1 mm as degree 1, increased mobility > 1 mm horizontally as degree 2, and degree 3 as severe mobility in both horizontal and vertical direction [26]. Two bitewing radiographs were obtained to estimate periodontal bone loss. All teeth but third molars were examined.

### Periodontal diagnosis

Periodontitis was diagnosed and classified using the 2017 World Workshop on the Classification of Periodontal and Peri-implant Diseases and Conditions [27]. In short, stage I periodontitis was diagnosed in the presence of interdental CAL of 1–2 mm at the site of greatest loss, PD 3–4 mm, radiographic bone loss affecting the cervical third of the roots, and no tooth loss due to periodontitis; stage II as the presence of interdental CAL loss of 3–4 mm, PD 4–5 mm, radiographic bone loss affecting the cervical third of the roots, and no tooth loss due to periodontitis; stage III as the presence of interdental CAL loss of ≥ 5 mm, PD > 5 mm, radiographic bone loss affecting the middle third of the roots, and ≤ 4 teeth lost due to periodontitis; stage IV as the presence of interdental CAL loss of ≥ 5 mm, PD > 5 mm, the radiographic bone loss affecting the middle third or more of the roots, and > 4 teeth lost due to periodontitis and in the absence of 10 or more occluding pairs of teeth. The extension of periodontitis was categorized as localized or generalized. Localized, meaning less than 30% of examined teeth fulfil the minimum criteria for the patients´ acquired stage of periodontitis and generalized, meaning 30% or more of the teeth coincide with the acquired stage of periodontitis. Agreement on case definition of subjects with unclear status was reached by discussion between the examiners and the reference examiner (KNL). Grading of periodontitis was not performed.

### Statistical analysis

Mean values and standard deviations were used to describe continuous variables. For categorical variables frequencies and percentages were used. Quantitative variables such as PD and CAL were sectioned into three distinct groups: 0–3 mm [1], 4–5 mm [2] and ≥ 6 mm [3]. The variables associated with MetS; HDL, triglycerides, serum glucose and blood pressure are categorical, and analysed based on the limit value for these variables used in the definition for MetS; NCEP ATP III. Data on furcation, tooth mobility, and radiographic bone loss are not reported due to the low number of positive observations.

For continuous variables at patient level, two-group t-test was used to test statistical differences, while chi-square tests were used for the categorical variables. Continuous variables were analysed using linear regression models reporting beta coefficients with 95% confidence intervals (95% CI). The categorical (dichotomous) outcome variables were analysed using logistic regression models, reporting odds ratios (ORs) with 95% CI. For tooth and site-specific outcomes variables, the regression analyses were performed using cluster robust variance estimates to adjust for multiple measures per individual. Stata version 17 (TX, USA) was used for the statistical analyses. P-values less than 0.05 were considered statistically significant.

## Results

A total of 52 subjects (1456 teeth/8736 sites) meeting study inclusion criteria were examined (Fig. 1). Fourteen were diagnosed as non-MetS (mean age 44.3 years, range 22–73 years) and 38 as MetS (mean age 49.1 years, range 26–66 years).

Characteristics and severity of periodontitis for included subjects are shown in Table 1. The non-MetS group included 10 females and 4 males, the MetS group 18 females and 20 males (p = 0.123). Subjects with and without MetS were similar in all characteristics, except elevated serum glucose (p = 0.025) and triglycerides (p = 0.004) mean values.

Total percentage subjects diagnosed with periodontitis was 78.6% in the non-MetS group vs.78.9% in the MetS group (p = 0.20). Five (35.7%) and six (42.9%) subjects in the non-MetS group were classified as stage I/stage II and stage III/stage IV (p = 0.25). In the MetS group the numbers were 16 (42.1%) and 14 (36.8%).

**Table 1:** Study sample characteristics on patient level

Variable	Non-MetS	N	MetS	N	P-value
Age in years	44.3 (15.6)*	14	49.1 (11.0)*	38	0.219
Sex		14		38	0.123
Male	4 (28.6%)		20 (52.6%)		
Female	10 (71.4%)		18 (47.4%)		
Ethnicity		14		38	0.928
Norwegian	13 (92.9%)		35 (92.1%)		
Other	1 (7.1%)		3 (7.9%)		
Smoking		14		38	0.753
Current smoker	2 (14.3%)		3 (7.9%)		
Former smoker	1 (7.1%)		4 (10.5%)		
Never smoker	11 (78.6%)		31 (81.6%)		
Snuff		14		38	0.748
Yes	2 (14.3%)		3 (7.9%)		
Former user	1 (7.1%)		2 (5.3%)		
No	11 (78.6%)		33 (86.8%)		
BMI (kg/m^2^)	42.4 (4.87)*	14	41.1 (4.02)*	38	0.315
Brushing teeth ≥ 2 daily	8 (57.1%)	14	23 (60.5%)	38	0.825
Use of interdental cleaning aids (floss, toothpick, ID-brush)	10 (71.4%)	14	28 (73.7%)	38	
HDL, female (mmol/L)	1.3 (0.18)*	10	1.3 (0.40)*	18	0.980
HDL, male (mmol/L)	1.2 (0.22)*	4	1.1 (0.23)*	20	0.232
BP (mm HG)		12		34	
Systolic	123.9 (39.4)*		126.6 (47.0)*		0.856
Diastolic	78.8 (24.8)*		75.4 (29.8)*		0.714
HbA1c (mmol/mol)	34.0 (3.46)*	14	38.8 (10.5)*	37	0.101
S-glucose (mg/dL)	5.00 (1.89)*	13	6.58 (2.26)*	38	**0.025**
Triglycerides (mmol/L)	1.29 (0.38)*	14	2.27 (1.19)*	38	**0.004**
CRP (mg/dL)	3.07 (5.54)*	6	3.71 (6.31)*	25	0.740
Stage of periodontitis		14		38	0.200
No periodontitis	3 (21.4%)		8 (21.1%)		
Stage I/Stage II	5 (35.7%)		16 (42.1%)		
Stage III/Stage IV	6 (42.9%)		14 (36.8%)		
Extent of periodontitis		14		38	0.252
Localised	6 (54.5%)		22 (73.3%)		
Generalised	5 (45.5%)		8 (26.7%)		

*=Mean (standard deviation), non-MetS = non-metabolic syndrome, *MetS = metabolic syndrome, BMI = body mass index, HDL = high density lipoprotein, BP = blood pressure, mm HG = millimetre of mercury, HbA1c = glycated haemoglobin, S-glucose = serum glucose, CRP = C-reactive protein, Smoking: current smoker (yes) former/never smoker (no)*

Table 2 presents the distribution of PD, CAL, and BoP. No differences in distribution of PD and CAL categories were observed between subjects with and without MetS (p = 0.94, both). In the non-MetS group, 29.8% of sites showed BoP, whereas the percentage in the MetS group was 23.5 (p = 0.048).

**Table 2:** The distribution of probing depth, clinical attachment level, and bleeding on probing in various categories for the non-metabolic syndrome (non-MetS) and metabolic syndrome (MetS) group at site level

Baseline			
	non-MetS	MetS	P-value
PD	n = 2352	n = 6384	0.942
0–3 mm	2 060 (87.6%)	5 587 (87.5%)	
4–5 mm	280 (11.9%)	758 (11.9%)	
≥ 6 mm	12 (0.5%)	39 (0.6%)	
CAL	n = 2352	n = 6384	0.942
0–3 mm	1 992 (84.7%)	5 397 (84.5%)	
4–5 mm	331 (14.1%)	917 (14.4%)	
≥ 6 mm	29 (1.2%)	70 (1.1%)	
BoP	n = 1492	n = 4015	**0.048**
No	1 049 (70.2%)	3 073 (76.5%)	
Yes	445 (29.8%)	942 (23.5%)	

PD = probing depth, CAL = clinical attachment level, BoP = bleeding on probing

The outcomes of unadjusted and adjusted logistic regression analyses for interdental PD (on site level), CAL (on site level), and stage III/stage IV periodontitis (on patient level) are shown in Tables 3 and 4, and 5. The unadjusted analyses showed that age had a significant impact on stage III/stage IV periodontitis (p = 0.008; Table 5).

**Table 3:** Odds ratio (OR) for having interdental probing depth (PD) ≥ 4 mm, at site level, when adjusted for variables associated with obesity and metabolic syndrome (MetS)

	Unadjusted		Adjusted for obesity related variables		Adjusted for obesity related variables and MetS	
	OR (95% CI)	p	OR (95% CI)	p	OR (95% CI)	p
Smoking vs. never smoking	1.07 (0.31–3.70)	0.917	1.05 (0.32–2.41)	0.941	1.54 (0.36–6.57)	0.556
Age	1.02 (1.00-1.05)	0.113	1.02 (1.00-1-04)	0.117	1.02 (0.99–1.04)	0.162
Females vs. males	1.07 (0.57–2.02)	0.837	1.10 (0.56–2.14)	0.788	1.05 (0.49–2.24)	0.901
BMI	0.97 (0.89–1.06)	0.538	0.98 (0.90–1.07)	0.724	0.99 (0.91–1.07)	0.763
CRP	1.01 (0.92–1.10)	0.907	1.00 (0.91–1.09)	0.997	1.00 (0.92–1.10)	0.951
HDL	0.77 (0.41–1.43)	0.405			1.05 (0.52–2.14)	0.890
Triglycerides	1.10 (0.58–2.08)	0.770			0.88 (0.42–1.88)	0.749
S-glucose	1.23 (0.66–2.29)	0.522			1.18 (0.65–2.14)	0.591
BP	1.68 (0.78–3.63)	0.187			1.87 (0.78–4.46)	0.161

BMI = body mass index, CRP = C-reactive protein, HDL = high density lipoprotein, S-glucose = serum glucose, BP = blood pressure

**Table 4:** Odds ratio (OR) for clinical attachment level (CAL) ≥ 4 mm at site level when adjusted for variables associated with obesity and metabolic syndrome (MetS)

	Unadjusted		Adjusted for obesity related variables		Adjusted for obesity related variables and MetS	
	OR (95% CI)	p	OR (95% CI)	p	OR (95% CI)	p
Smoking vs. never smoking	1.03 (0.29–3.65)	0.963	1.06 (0.32–3.52)	0.923	1.48 (0.36–6.19)	0.588
Age	1.02 (1.00-1.05)	0.087	1.02 (1.00-1.04)	0.088	1.02 (0.99–1.04)	0.124
Females vs. males	0.97 (0.53–1.77)	0.913	0.98 (0.52–1.87)	0.960	0.93 (0.46–1.91)	0.852
BMI	0.98 (0.90–1.06)	0.549	0.99 (0.91–1.07)	0.800	0.99 (0.92–1.07)	0.833
CRP	1.00 (0.92–1.09)	0.935	1.00 (0.92–1.08)	0.969	1.00 (0.92–1.09)	0.976
HDL	0.70 (0.38–1.30)	0.262			0.91 (0.46–1.81)	0.793
Triglycerides	1.12 (0.61–2.05)	0.716			0.89 (0.44–1.83)	0.759
S-glucose	1.17 (0.64–2.14)	0.601			1.14 (0.65–2.02)	0.646
BP	1.65 (0.78–3.46)	0.189			1.66 (0.73–3.77)	0.231

BMI = body mass index, CRP = C-reactive protein, HDL = high density lipoprotein, S-glucose = serum glucose, BP = blood pressure

When adjusted for obesity related variables and MetS, the effect of age on stage III/stage IV periodontitis, persisted to be significant (Table 5; p = 0.006 and p = 0.002, respectively). The effect of age on PD and CAL continued to be non-significant (Tables 3 and 4).

**Table 5:** Odds ratio (OR) for having stage III/stage IV periodontitis, at patient level, when adjusted for variables associated with obesity and metabolic syndrome (MetS)

	Unadjusted		Adjusted for obesity related variables		Adjusted for obesity related variables and MetS	
	OR (95% CI)	p	OR (95% CI)	p	OR (95% CI)	p
Smoking vs. never smoking	1.07 (0.16–7.19)	0.941	0.30 (0.00-36.38)	0.626	0.34 (0.00-63.78)	0.687
Age	1.09 (1.02–1.17)	**0.008**	1.15 (1.04–1.26)	**0.006**	1.15 (1.05–1.26)	**0.002**
Females vs. males	1.22 (0.39–3.79)	0.728	4.64 (0.68–31.67)	0.117	7.34 (0.77-70.00)	0.083
BMI	0.99 (0.87–1.13)	0.889	1.08 (0.87–1.34)	0.489	1.04 (0.82–1.32)	0.734
CRP	0.99 (0.87–1.13)	0.889	1.04 (0.84–1.29)	0.691	1.04 (0.85–1.29)	0.690
HDL	1.01 (0.90–1.14)	0.812			5.75 (0.36–92.55)	0.218
Triglycerides	0.56 (0.16–1.94)	0.357			1.17 (0.18–7.78)	0.868
S-glucose	1.86 (0.58–5.94)	0.296			0.68 (0.12–3.95)	0.672
BP	0.84 (0.27–2.61)	0.758			9.50 (0.27-339.51)	0.217

BMI = body mass index, CRP = C-reactive protein, HDL = high density lipoprotein, S-glucose = serum glucose, BP = blood pressure

## Discussion

### Summary and comparison with other studies

In a sample of obese subjects with a mean BMI of 41.5 kg/m^2^, the occurrence of periodontitis was not found to be associated with MetS. The data analyses revealed a higher percentage cases with severe periodontitis in the non-MetS group (stage III/stage IV: 42.9%) compared with the MetS group (stage III/stage IV: 36.8%). Similar distributions of PD and CAL were observed in the two groups. Interestingly, a significantly higher percentage BoP was observed in the non-MetS compared with the MetS group. For staging of periodontitis, the effect of age appears to be significant both for obesity related variables and MetS.

We hypothesized that MetS would promote a more severe periodontal conditions in obese subjects. However, the prevalence of periodontitis was similar (78.6% in non-MetS vs. 78.9% in MetS), the non-MetS group exhibiting a slightly higher prevalence severe periodontitis (42.9% in non-MetS vs. 36.8% in MetS). Moreover, BoP was more prevalent in the non-MetS group (29.8% in non-MetS vs. 23.5% in MetS; P = 0.048). The questionnaire, however, exploring personal oral hygiene routines disclosed comparable findings (73.7% in the MetS vs. 71.4% in the non-MetS group).

A study on the prevalence and severity of periodontitis in a Tromsø, Norway, normal-weight population (mean age 47.3 years, range 20–79 years), reported a prevalence of 49.5% [28]. Despite similar study mean age and rather high prevalence of periodontitis possibly caused by less frequent use of dental services in the northern regions of Norway, the present findings both in the non-MetS and the MetS group appeared to be substantially higher (> 78%). The production and systemic distribution of the pro-inflammatory cytokines in obese subjects vary depending on the subtype of adipose tissue, with visceral adipose tissue being the subtype most often associated with development of comorbidities and systemic adverse effects [29, 30]. Low-grade systemic inflammation is most likely the causal association between obesity and periodontitis and thus, partly explaining the high prevalence of periodontitis in the present study [31, 32].

A study from northern Finland [33] investigated the association between periodontal pockets and obesity defined by BMI and WC. The participants were categorised at the age of 31 and 46 years, while periodontal examination was undertaken at the age of 46. This design allowed the authors to investigate the long-term effects of obesity. Both parameters showed an association between periodontitis and long-term obesity, but periodontitis was more strongly associated with WC than with BMI. The article suggests that BMI is an inferior parameter for obesity compared with WC because it does not consider the distribution of fat throughout the body. WC captures central obesity and therefore might be a more appropriate indicator for the low-grade systemic inflammatory effects of visceral body fat. Overall, this indicates that both obesity and central obesity may exert harmful effects on periodontal health beyond what is observed in cross-sectional studies.

The association between prevalence of periodontitis and MetS was investigated in Vietnamese subjects allocated to an MetS (mean age 57.8/mean BMI 28.3 kg/m^2^) or a non-MetS (mean age 58.2/mean BMI 27.0 kg/m^2^) group [34]. Subjects with more severe periodontitis presented with a higher risk of MetS. A significantly higher percentage severe periodontitis was thus observed in the MetS vs. that in the non-MetS group (37.9% vs. 19.4%). The present study reported high prevalence of severe periodontitis in both groups (42.1% vs. 35.7%), and with non-significant intergroup difference. With a lower mean age (49.1 years in the Mets vs. 44.3 years in the non-Mets) and despite lifestyle differences between western culture Norway and south-eastern culture Vietnam making the comparison difficult, the severity of periodontitis in the present study might be expected to be at the same level or less than in the Vietnamese study.

A Brazilian case-control study also reported a risk association between MetS and periodontitis [35]. The prevalence of periodontitis was 54.6% in the MetS group and 35.6% in the control group. Study subjects were categorized based on BMI ≤ 25 kg/m^2^ and > 25 kg/m^2^. However, no information on mean group BMI was available. The overall prevalence of periodontitis in our study groups approximated 79%. This may indicate that exceeding a certain level of overweight/obesity the association between MetS and periodontitis becomes less significant as the dominating impact of obesity relates variables undermines the detrimental effect of other systemic factors.

The regression analysis showed a tendency toward significant impact of gender on the development of stage III/stage IV periodontitis when adjusting for MetS variables (p = 0.083). Age had a significant effect on the prevalence of stage III/stage IV periodontitis. The effect of age might be expected as periodontitis is an irreversible, cumulative disease [36, 37]. As the non-MetS group is rather heterogeneous, including subjects with high BMI who also have other elements of MetS comorbidities, the lack of observed deterioration from the MetS was not unexpected. Higher mean values for triglyceride and serum glucose were measured for the MetS group. For the other criteria defining MetS, the non-MetS group showed relatively high values as well, yet, not meeting the requirements for a MetS diagnosis [9]. In addition, some subjects in the MetS group were medicated for health-related problems such as high blood pressure, and thereby distorting general health parameters. In other words, the combination of a non-MetS group with borderline requirements for MetS and a MetS group receiving medical treatment for comorbidities, may lead to non-significant risk indicator differences between the groups. In addition, a more recent definition of MetS using lower cut-off values for WC and fasting glucose [6] may result in a higher MetS prevalence possibly attenuating the association between periodontitis and MetS [38].

### Methodological challenges and strengths

The unbalanced distribution of subjects within the two groups, with a lower number of subjects included in the non-MetS group, might undermine the statistical power of the study. In the present study, staging and grading were primarily based on clinical measures such as CAL, PD, furcation involvement, and bitewing radiographs. Registration of plaque would give a more nuanced understanding of all clinical parameters and particularly BoP. The rather high mean BMI of our study sample may undermine the likelihood of detecting intergroup differences. Only about 15% of the obese population will not develop obesity related co-morbidities [39]. In addition, a 5-month lifestyle intervention course may affect the interpretation of the study outcomes. The use of calcium channel blockers to treat hypertension, angina pectoris, or cardiac arrhythmias, is a widely known risk factor for development of drug induced gingival overgrowth, which can affect the gingival conditions and influence the development/progression of periodontitis [40–42]. Due to the low number of study subjects, the estimates from the adjusted regression analyses should be interpretated cautiously. The interpretation of study outcomes without a satisfactory matched control group of normal-weight subjects needs to be made with caution. Future studies evaluating the potential association between obesity and periodontitis and the influence of Mets require inclusion of a normal-weight control group and a non-MetS group with comparable levels of obesity and absence of MetS comorbidities.

The strength of this study is the full-mouth periodontal evaluation, and the periodontal examination performed by only one examiner with high ICC values, improving the disease detection accuracy. The sociodemographic, oral hygiene habits, laboratory and clinical data were rather comprehensive and relevant.

## Conclusions

In the current sample of obese subjects, periodontitis occurred independently of MetS. The data analyses showed that a higher percentage cases with severe periodontitis was found in the non-MetS group (stage III/stage IV: 43%) compared with the MetS group (stage III/stage IV: 37%). A significantly higher percentage of BoP was detected in the non-MetS group. For stage III/stage IV periodontitis, the effect of age appears to be significant both for obesity related variables and MetS. Apparently, when a certain BMI level is reached the suggested association between MetS and periodontitis seems to be inferior due to the dominating impact of obesity related variables possibly undermining the significance of other systemic factors.

## Data Availability

The datasets used and analysed during the current study and coding are available from the corresponding author on reasonable request.

## Abbreviations

BMI: Body mass index
MetS: Metabolic syndrome
HUH: Haukeland University Hospital
PD: Probing depth
BoP: Bleeding on probing
CAL: Clinical attachment level
HDL: High-density lipoprotein
TG: High triglyceride
CRP: C-reactive protein
ICC: Intraclass correlation coefficients
WC: Waist circumstance
BP: Blood pressure
